# Failure to thrive in infant secondary to congenital colonic stenosis: a case report

**DOI:** 10.1093/jscr/rjae766

**Published:** 2024-12-05

**Authors:** Daniel Jose Garcia, Mohamad Hamade, Li Lin, Matias Matias, Armaan Sobhan, Mario Zaritsky, Chad Thorson

**Affiliations:** Department of Surgery, University of Miami, PO Box 016960 (C203), Miami, FL 33101, United States; Department of Radiology, University of Miami, 1150 NW 14th St #511, Miami, FL 33136, United States; Department of Pathology, University of Miami, 1120 NW 14th St, #C1403, Miami, FL 33136, United States; St. Georges University School of Medicine, University Centre Grenada, West Indies, Grenada; St. Georges University School of Medicine, University Centre Grenada, West Indies, Grenada; Department of Radiology, University of Miami, 1150 NW 14th St #511, Miami, FL 33136, United States; Department of Surgery, University of Miami, PO Box 016960 (C203), Miami, FL 33101, United States

**Keywords:** congenital colonic stenosis, failure to thrive, surgery, pediatric surgery, laparotomy, obstruction, hemicolectomy, colectomy, plexus, ganglion

## Abstract

Congenital colonic stenosis (CCS) is a rare cause of intestinal obstruction, most commonly presenting in the neonatal period. We present a case of delayed CCS and describe the diagnostic challenges experienced. A 16-week-old female patient presented with persistent failure to thrive associated with signs of intestinal obstruction. Prior encounters included investigation for pyloric stenosis, gastrointestinal pathogens, acid reflux, and cow milk allergy, with no to little improvement in symptoms. Abdominal imaging showed bowel dilation with possible colonic obstruction while excluding malrotation. Exploratory laparotomy revealed abrupt reduction in caliber of the mid/distal transverse colon and extreme luminal narrowing, consistent with colonic stenosis. Extended right hemicolectomy and anastomosis resulted in returned bowel function and appropriate weight gain in follow-up. Though rare, CCS should be considered in cases of partial or subacute intestinal obstruction throughout the first year of life. Inconclusive clinical and imaging results may support exploratory laparotomy after excluding differential diagnoses.

## Introduction

Congenital colonic stenosis (CCS) is a rare disorder in infants and is commonly manifested by acute or subacute intestinal obstruction. Incidence of CCS is lower than other types of congenital colonic atresia, with a rate of less than 0.25 per 10 000 births [1, 2]. While CCS typically presents during the neonatal period, it has been described in older infants and young children [3–5]. In contrast with other pediatric bowel obstructions, diagnostic imaging workup for CCS can be unreliable, with diagnosis achieved only at the time of surgery in many cases [3, 6]. This case report describes our experience with CCS beyond the neonatal period, complicated by nonspecific gastrointestinal (GI) symptoms and nondiagnostic imaging results.

This manuscript was prepared following the CARE guidelines (https://www.care-statement.org).

## Case report

A 16-week-old female patient was admitted to our facility with failure to thrive associated with decreased oral intake, vomiting, abdominal distension, and absolute constipation. She was born by spontaneous vaginal delivery at the 39th week of gestation, with reported maternal COVID-19 infection during pregnancy. No maternal smoking or use of vasoconstrictor agents was reported. The patient’s weight at birth was 44th percentile, and she was initially discharged without complications. She was hospitalized at 3 days old for severe jaundice with normal liver function tests and was discharged after successful treatment with triple phototherapy.

Vomiting and poor weight gain were noted at 2 weeks of age, at which time the patient was exclusively breastfed. Ultrasound excluded pyloric stenosis, and cow milk-based infant formula was introduced to supplement breast milk. At 11 weeks old, she presented again with failure to thrive (weight at 11th percentile), low oral intake, reflux symptoms, and chronic vomiting and diarrhea. In the previous 4 days, she had exhibited nonbloody, nonbilious projectile vomiting with every meal; stool was yellow-green with no blood or mucus. The patient appeared dehydrated, and their weight was in the third percentile. Ultrasound was again negative for pyloric stenosis. Laboratory investigations revealed band cell count of 10% and C-reactive protein level of 3 mg/l. GI pathogens panel revealed *Escherichia coli*. The patient was started on azithromycin and famotidine. She was discharged after 3 days with instructions to avoid cow milk protein.

**Figure 1:**
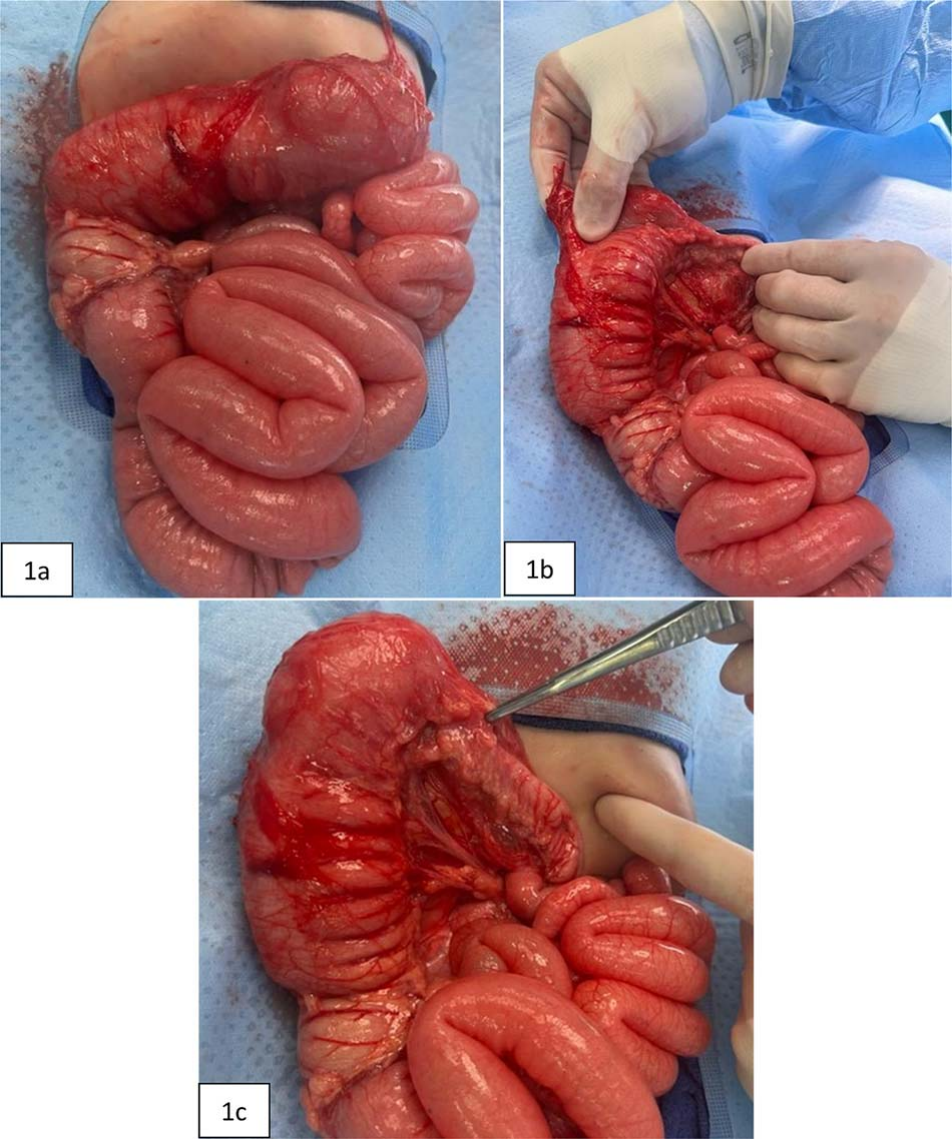
Intraoperative pictures taken prior to extended right hemicolectomy. Figure 1(a) shows the ascending colon and dilated transverse colon, along with diffuse enlargement of small bowel. Figure 1(b) demonstrates the difference in diameters of the proximal versus distal transverse colon. Figure 1(c) highlights the thickened, narrowed lumen of the distal transverse colon.

**Figure 2:**
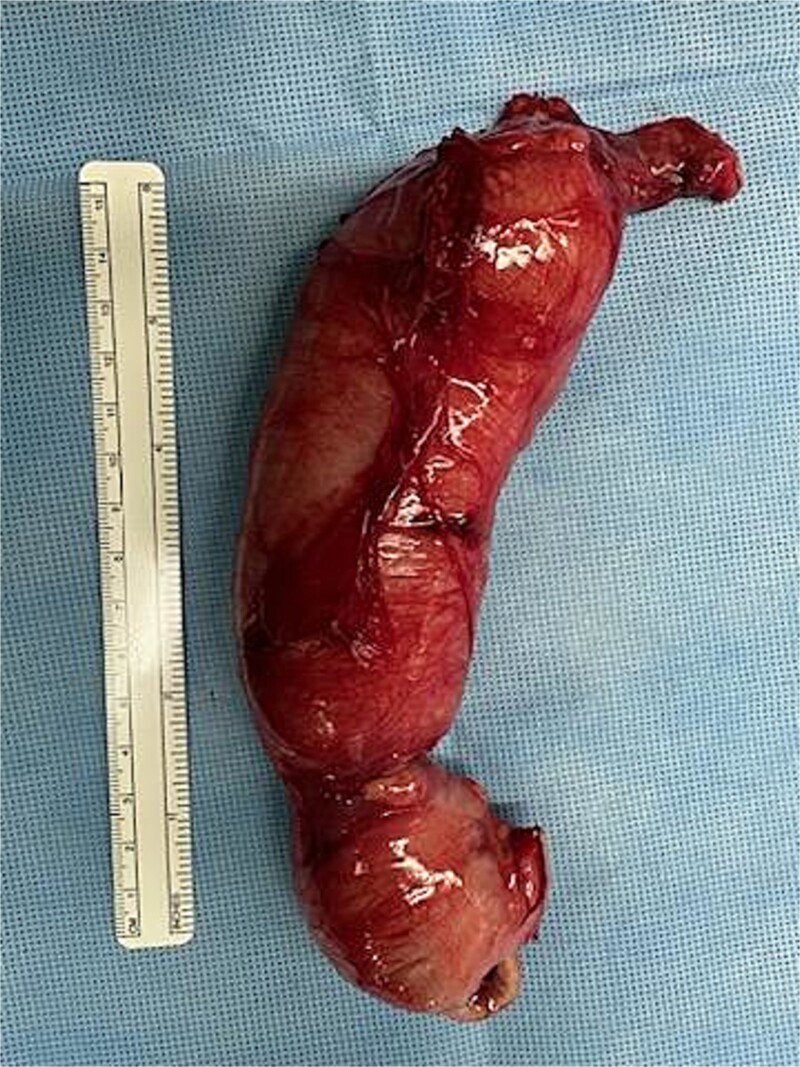
Postoperative image taken of the resected bowel. The south end contains the proximal large bowel (ileocecal valve and the cecum), and the distal portion shows the terminal end of the atretic transverse colon.

**Figure 3:**
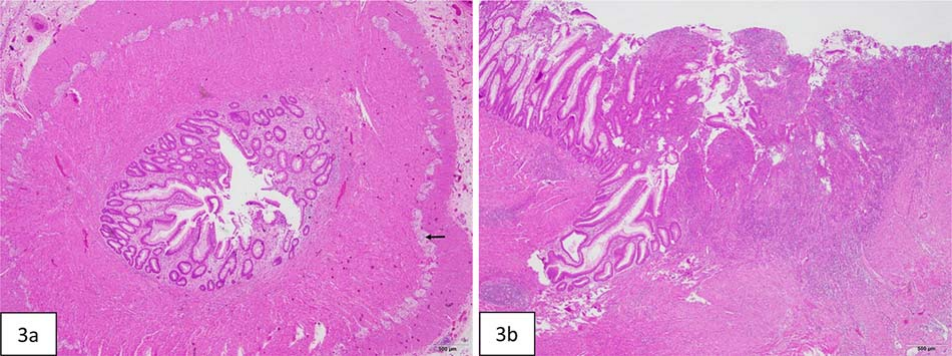
Histopathologic evaluation of the resected bowel. (a) Cross-section of stenotic colon shows severely narrowed lumen. Ganglion cells are present in Auerbach plexus (arrow). (b) Mucosal ulceration (right side of figure) in the adjacent colon proximal to the stenosis.

At 16 weeks of age, the patient presented again with minimal improvement of previous complaints and absolute constipation. Bowel sounds were absent, abdomen was extremely distended, and weight was fourth percentile. Abdominal radiograph showed distension of right and mid-abdominal small intestine as well as severe gastric distention. Upper GI radiography series excluded malrotation but revealed distended transverse colon. Contrast enema showed possible obstruction at the splenic flexure. Abdominal ultrasound suggested an enteric duplication cyst with the gut signature sign, localized to the hepatic flexure of the ascending colon. However, exploratory laparotomy ultimately revealed an abrupt reduction in caliber of the mid/distal transverse colon with extreme luminal narrowing (Fig. 1), consistent with colonic stenosis. Extended right hemicolectomy and anastomosis of distal colon with terminal ileum were performed (Fig. 2). Histopathologic analysis showed the presence of ganglion cells in Auerbach’s plexus and mucosal ulceration adjacent to the stenosis (Fig. 3). The patient was discharged following the return of bowel function on the third day post-surgery, and appropriate weight gain was noted in subsequent follow-up.

## Discussion

CCS is a potentially life-threatening malformation of the intestinal wall typically presenting in the neonatal period with failure to pass meconium, abdominal distension, and/or bilious vomiting. It has been classified as a subtype of colonic atresia [1, 7]. Due to its rarity, the incidence of CCS is difficult to ascertain; as of 2023, there were 35 reported cases in the literature [3].

While acquired colonic atresia can be caused by scarring and vascular disruptions induced by infection or injury to the colon (such as necrotizing enterocolitis), the origins of congenital cases are unclear. Mesenteric vascular insufficiency and failure of intestinal recanalization during the solid stage of development have both been proposed to lead to congenital atresia/stenosis [8–10]. Other co-occurring factors have included congenital varicella and maternal exposure to nicotine, cocaine, and vasoconstrictor drugs [11–13]. Hirschsprung disease and malrotation may result in strictures that mimic CCS [3, 14–16].

While in the reported case, there was no evidence of *in utero* exposure to varicella, nicotine, cocaine, or vasoconstrictor drugs, there was maternal infection with COVID-19 during pregnancy. Despite the associated hypercoagulable state and hypothetical link to fetal vascular anomalies, COVID-19 infection has not been documented to increase the incidence of intestinal atresia [17]. Unconjugated hyperbilirubinemia, which has been shown to be an early symptom of congenital bowel obstruction [18], was noted during the patient’s first week of life. However, at the time of neonatal hyperbilirubinemia, there were no signs of decreased gut motility, and stool and feeding patterns were normal.

Approximately one-third of congenital colonic obstructions in the United States are currently detected by ultrasound screening in the prenatal period [19]. Of those that remain undetected prior to birth, the majority of cases are present in the first few days of life. Later presentation in infancy and early childhood has been described as well; [3–5] delayed presentation may occur due to acute or gradual loss of patency in the stenotic section.

Despite standard laboratory investigations, radiography, and ultrasound, colonic stenosis was not diagnosed prior to surgery. This experience is echoed in previous reports, where symptoms were nonspecific as to the location of the obstruction and imaging was inconclusive. Though a staged approach with initial stoma creation and delayed repair may be appropriate, our case was managed by extended right hemicolectomy and anastomosis, and the patient recovered gut function and weight gain.

## Conclusion

Though rare, CCS should be considered and investigated in cases of partial or subacute intestinal obstruction throughout the first year of life. Inconclusive clinical and imaging results may support exploratory laparotomy after excluding differential diagnoses.
